# Devolution after Empire

**DOI:** 10.1093/ojls/gqag010

**Published:** 2026-04-20

**Authors:** Ewan Smith

**Keywords:** devolution, empires, constitutional theory, constitutional law

## Abstract

This article questions the claim that the UK is a unitary state. It argues that the UK was, and perhaps is, an empire. Empires are states in which questions of subsidiarity are inescapable and ongoing. Like unitary states, they have a supreme central government. But unlike unitary states, empires conceal multiple constitutional orders, overlaid with the veneer of sovereignty. The nature of the UK territory was ambiguous under empire. It still is. The UK is not a state where all parts bear the same relationship to the centre; rather, the constituent parts of the UK bear separate relationships to the centre. The article explores how imperial structures continue to influence that territorial settlement. The UK constitution ultimately resolved large-scale problems of subsidiarity in a global empire. It remains to be seen whether that same structure can solve smaller problems.

## 1. Introduction

Many people describe the UK as a ‘unitary state’.^1^ Others say it used to be a unitary state, but since devolution they have not been sure.^2^ So the story goes, the UK can ‘be seen as one of the new nations which arose from the dissolution of one Empire’^3^ but with ‘not the smallest element of federation’.^4^ Some say, when we devolved power to Scotland, Wales and Northern Ireland, we put down that old constitution and took up a new, more federal one.^5^ But if the postwar UK constitution was unitary, when exactly was that constitution in force?

In 1900, Britain was an empire. In 1931, the dominions were granted self-government.^6^ In the decade following Harold MacMillan’s ‘Winds of Change’ speech, all but two of the UK’s African colonies were granted some form of independence.^7^ In 1968, Edward Heath committed the Unionist Party to home rule.^8^ The following year, the Labour government launched a royal commission charged to bring forth proposals for devolution. In 1982 (four years after those proposals were put on ice), the UK Parliament granted plenary legislative power to Canada, then later Australia.^9^ In July 1997 the UK published the White Paper *Scotland’s Parliament*^10^ three weeks after it returned Hong Kong to China. In 2025, it acknowledged Mauritian sovereignty over the Chagos Islands.^11^ It is hard to identify a moment, in this sequence of events, when the imperial constitution ended and the unitary one began.

This article questions the claim that the UK is, still less was, a unitary state. It argues that it was, and perhaps is, an empire. Unlike unitary states, empires are not really governed as a single entity. They sometimes have two constitutional orders: one for the metropole and one for everywhere else. More often, they are characterised by many overlapping constitutional orders. The constitutional order that prevails in the UK today reflects that earlier, multipolar tradition. This article explains the consequences.

The dismantling of empire abroad and the construction of home rule at home are continuous and connected processes.^12^ Unlike France, there is no moment when the UK ceased to be a seconde empire and became a troisième republique. But perhaps the UK was unitary in a different way. Perhaps the metropole^13^ was always a unitary state, while the empire was not. Here, the key challenge has to do with *where* the empire ended, not *when*. But ‘where is the UK’ is a harder question that it might seem. Unlike France or Rome, our metropole and our empire have always overlapped. Northern Ireland is a core part of the UK’s core territory. It is also a settler colony, ruled directly by the UK government between 1972 and 1998 under exceptional legal circumstances, whose devolved government has been suspended six times since then. We might ask whether the Crown Dependencies are part of this core or part of the periphery. Politically, if not constitutionally, the Isle of Man is closer to Great Britain than Northern Ireland. We might ask whether British Overseas Territories are British, foreign or other.

A more promising argument is that the UK is a special sort of unitary state: a state that enjoys a special unity, a unity that encompasses the diversity of empire. The UK and its dependent territories arguably form one realm under one undivided Crown. That realm is governed by a sovereign Parliament with unlimited power to legislate for the whole of the UK territory. Its head is a monarch, whose reign connects the realm. Perhaps it is the presence of these supreme, central constitutional structures, not the homogeneity of the metropole, that make the UK a unitary state. But if the UK is everything under the Crown and Parliament, then perhaps our constitution includes Commonwealth territories. We might ask again whether this unity is real or notional?

The question matters because the answer has direct implications. Twenty-first-century cases in the UK’s apex court turn on questions such as ‘did the minister make the decision for the Crown of the UK or the Crown of the South Georgia and Sandwich Islands’.^14^ It is not easy to answer this question in a unitary frame of reference. It also has indirect implications. We cannot understand our present constitution unless we understand its continuity with our past. From the Union until the Partition of India, devolution in Britain was global, and the basic model was imperial. In 1776, some colonies disagreed with London about the right measure of subsidiarity and that disagreement was not solved constitutionally. From 1869, when the Irish Church was disestablished, until the Statute of Westminster in 1931, home rule was arguably the central question in UK politics. Arguably it still is.

Before we go further, it is important to acknowledge that the claim that the UK is ‘imperial’ comes freighted with political baggage. Allegations of imperialism are frequently deployed in arguments between nationalists and unionists in the UK, often in an inflammatory fashion. The intention here is not to amplify this rhetoric but to disarm it, by clarifying it. Vague allegations of imperialism circulate precisely because our understanding of imperialism is vague. A generation ago, Neil MacCormick argued that the UK is ‘missing any tradition of articulated reflection upon the nature of states’. It ‘evolved from an English feudal monarchy [and] became a state without avowing its changed character through a formal constitution’.

[B]y and large, legal and constitutional parlance in the United Kingdom does not make much use of the term ‘state’ as a technical term … What elsewhere is treated as a unitary acting state is in the United Kingdom discussed through doctrines about ‘the Crown’ or occasionally the ‘Executive’, and about the prerogatives of the Crown, about Parliament … and its privileges, and its omnipotence or indeed ‘sovereignty’.^15^

If we are to engage in ‘articulated reflection’ on the nature of the UK, that reflection leads us straight to empire.

It is necessary to say a little more about the terms ‘empire’, ‘devolution’ and ‘unitary state’. Nothing important turns on the exact definition of empire or devolution used here. This article does not make claims about empires in general, but about one empire in particular. Nor does it seek to make general claims about the nature of devolution. Stephen Howe says an empire is

a large political body which rules over territories outside its original borders. It has a central power or core territory—whose inhabitants usually continue to form the dominant ethnic or national group in the entire system—and an extensive periphery of dominated areas.^16^

Howe also highlights one important feature of empire which will be relevant to the argument here. Empires:

always involve a mixture of direct and indirect rule. The central power has ultimate sovereignty, and exercises some direct control, especially over military force and money-raising powers, in all parts of its domain. But there will usually be some kind of decentralized, ‘colonial’, or ‘provincial’ government in each of the empire's major parts, with subordinate but not trivial powers of its own.^17^

In turn, the Kilbrandon Commission defined devolution as ‘the delegation of central government powers without the relinquishment of sovereignty’.^18^ Anthony Bradley and Keith Ewing added that devolution ‘can mean what the respective parties to it need and want it to mean at any given time’.^19^ As we will see, by placing legal sovereignty in the foreground, the architects of the devolution settlement created a measure of ambiguity about the relationship between the parties, because they thought the ambiguity was a constructive one.

Unlike these two concepts, the argument does turn on the idea of a unitary state. In general, we can define the unitary state either in terms of something that is present or in terms of something that is absent. The thing that is present is usually a centralising legal sovereignty. On the first page of his monograph on the UK constitution, Nick Barber notes that, on one account, parliamentary sovereignty ‘required that the UK be a unitary state … On a unitary conception of the UK, the UK Parliament in Westminster retains its supremacy, able to override the decisions of devolved assemblies or remove the devolution settlement altogether’.^20^

In contrast, the thing that is absent is usually a legal and constitutional pluralism. Signe Rehling Larsen refers broadly to federations as ‘political union[s] of states founded on a constitutional compact’.^21^ Kenneth Wheare, the pre-eminent scholar of British imperial federalism, describes a system in which ‘each level of government has the final decision about some matters’.^22^ In contrast, in a unitary state, only one level of government has the final decision about all matters. Another way to pose this question is to ask if the constitution is singular or plural. If there is one regime in force in all places at all times, then the state is unitary; if there is more than one, then (at least in this sense) it is not.^23^

Not everyone describes the UK as a ‘unitary state. In *Public Law and Democracy in the United Kingdom and the United States of America*, Paul Craig charts a distinctive British pluralism in the work of Harold Laski, John Neville Figgis and Frederic Maitland.^24^ In turn, James Mitchell^25^ draws on work by Gaspare Ambrosini and Bill McKenzie.^26^ He argues that the UK is not a unitary state but a union state, a state with a number of unions that evolved over many decades. Neil Walker describes the UK as a pluralist state, a state whose federalism is ‘a graduated state of affairs’ rather than something conceived in ‘either/or terms’,^27^ a conclusion that echoes the work of Neil MacCormick. This article draws on these themes. But it is striking that empire is, at best, a minor theme in this postwar scholarship, as if imperial pluralism and metropolitan pluralism were separate ideas. As we will see, the answers to many modern constitutional questions come out differently if we ask the question with empire in mind.

Empire ought to be a central theme in this literature for at least two reasons, one theoretical and one practical. First, our constitution is presented to us through ideas forged by imperialists at the apex of the imperial period. These ideas masked the various constitutional regimes in force in the UK, in part because this was thought to be conducive to imperial unity. The theory of the UK constitution inevitably responds to these ideas, and they need to be addressed in their context.

Second, empires are states in which practical problems of subsidiarity are inescapable and ongoing. They maintain the guise of unitary governance, but in reality they embrace more than one legal order. Empires can never escape problems of subsidiarity: at best, they buy time in which to solve them durably. Imperial structures are unstable because they amplify both centrifugal and centripetal forces. The UK’s long-run constitutional history suggests that imperial devices such as sovereignty and the Crown are not stable solutions.

There is one further principle at play here which cannot be overlooked. Subsidiarity is a principle of justice. It demands that decisions be made as close as possible to the people who are affected by them, by decision makers who are accountable to those people. Paolo Carozza says subsidiarity requires:

larger communities to protect the legitimate autonomy of smaller communities, to provide them with the assistance … needed to fulfil their ends, and to coordinate and regulate their activities within the common good of the larger community, of which they are a part and which is also necessary to the flourishing of their individual members.^28^

Given the scope of this article, I cannot develop and defend a full account of subsidiarity, but subsidiarity is one yardstick by which I evaluate the constitutional structures explored here.

The point of this article is to consider the territorial constitution in light of empire. It begins, in section 2, by placing the central operating ideas of the UK constitution in their imperial context. Section 3 takes those conclusions and considers what they tell us about our present constitutional order. The shape and nature of the territory of the UK remains an area of particular complexity. We often try to explain that order with vague reference to the place where the ‘normal’ rules apply. But it can hard to say exactly where that place might be.

Decolonisation was neither smooth nor bloodless. Nonetheless, it ultimately managed to resolve very-large-scale questions of subsidiarity in a global empire. It remains to be seen whether those same constitutional structures can resolve small-scale questions of subsidiarity in a supposedly unitary state.

## 2. The Constitution on Which the Ink Never Dries

The familiar lines of the UK constitution—a flexible constitution which combines legal and political rules and which functions through the integration of parliamentary sovereignty and the rule of law—were drawn at the apex of the Age of Empires. This section is focused on that account of the constitution, which emerges at the turn of the 20th century. It draws two central conclusions, beginning with the questions posed in the introduction. First, it asks how many constitutional orders overlap in an empire, and considers three possible answers: one, two and lots. It argues that the final answer—lots—is the most convincing. To understand why, we first need to understand why many people thought the answer was both ‘one’ and ‘lots’ at the same time. Second, the section considers parliamentary sovereignty in its imperial context. It asks why Dicey thought the imperial Parliament retained ‘absolute sovereignty throughout every part of the British Empire’ and yet had also ‘renounced’ the ‘right’ to tax the colonies. It then asks what the answer tells us about sovereignty.

### A. Flexibility

James Bryce argued that the UK required an unusual constitution, one in which political authority was centralised. He distinguished between ‘flexible constitutions’ and ‘rigid constitutions’. Flexible constitutions ‘have elasticity, because they can be bent and altered in form while retaining their main features’.^29^ Rigid constitutions are hard and fixed. Flexible constitutions are jealous of constituent power and juridical entrenchment. They rest on convention. They are, in the modern idiom, ‘political’ constitutions.

The key examples of flexible constitutions are Rome and England. These constitutions ‘are suitable to a State which is expanding its territory and taking in other communities whether by conquest or by treaty’.^30^ They have only one form of legislative authority and no superior law. Sovereign power is ‘lodged in an assembly which can, on occasion, act with extraordinary promptitude’.^31^ These constitutions leave the very idea of the state cast in soft focus:

[T]here was in strictness no Roman Constitution [as t]here is no British Constitution … That which we call the Constitution of the Roman State, that which we now call the Constitution of the United Kingdom, is a mass of precedents, carried in men’s memories or recorded in writing, of dicta of lawyers or statesmen, of customs, usages, understandings and beliefs bearing upon the methods of government …^32^

Bryce thought this flexibility and its centralising sovereignty were conducive to empire. They enabled the constitutional order to expand across the globe with limited friction. But this is only one view. By Bryce’s account, empire was the extension of an essentially English model. On another view, empire accommodated multiple of models. Dylan Lino, for example, underlines the heterogeneity of the rules and institutions of empire and the notional centrality of Parliamentary Sovereignty.^33^ This enabled ‘the deployment of different forms of rule suited for the different peoples. We can see now, that the flexibility of the UK constitution is both an artefact of empire, and connected to the question ‘how many constitutions does the UK integrate?’ So what was the constitutional order of the British Empire? Was it singular, dual or plural?

In his inaugural lecture, David Feldman asked how many constitutions the UK has. The options presented are none, one or several.^34^ Feldman quickly dismisses the case for ‘none’, but he prevaricates between one and several, noting that ‘even before devolution there was a distinctive view of the constitution in Scotland’. It is striking that Feldman’s menu does not include ‘two’. If the empire was not unitary, then it may have been a dual: a state characterised by the separation of empire and metropole? Josep Fradera distinguishes ‘imperial’ and ‘colonial’ constitutions. Imperial constitutions had a single constitutional order. Colonial constitutions had one constitutional order for the metropole and another for the colonies.^35^ In turn, Bryce drew a sharp distinction between the metropole and the empire. The inhabitants of the UK were represented by the UK Parliament. The inhabitants of the empire were not.

One reason why the question is important is because it can tell us what is left when empire finally recedes. If there were two constitutions—one home, one abroad—then perhaps the home nations formed a unitary state *throughout* the imperial period. On this account, after empire, a unitary state remained in the UK metropole, and we can ignore what happened in India, Jamaica, South Africa and so on. In contrast, if an imperial constitution was in force at home throughout the imperial period, we might still expect to find its remnants.

The most influential theory of the constitutional order developed in this period is Dicey’s. Dicey refers liberally to empire. He did not describe the UK as a unitary state, but he did describe it as having a ‘unitary constitution’. One way to read Dicey’s claim that the empire has a unitary constitution is that it is equivalent to Bryce’s claim that empire was a simple extension of the English unitary state. But this view is hard to sustain and Dicey does not quite sustain it. In the Introduction, he thunders that the suspension of ordinary law ‘is unknown to the law of England’. He whispers, in a footnote, that this statement ‘has no reference to’ empire, which is to say, no reference to the vast majority of imperial subjects.^36^

Dicey develops an account of the imperial constitution, couched in terms of broad principles, but hedged by local exceptions. In *The Forest Ranger*, Herbert Kaufman describes an bureaucracy in which isolated officials solve problems at many days’ journey from headquarters.^37^ In this unpredictable environment, one cannot secure consistent action by detailed fixed rules. The officials are not going to consult a manual. What is needed is coordination by general, guiding principles. Dicey’s model of the imperial constitution is of this type. It provides general, flexible guidance in circumstances where uniform, specific guidance would be impractical. Lino describes this as a ‘malleable, functional [definition] of a constitution—open to incorporating varied and multiple governing institutions, territories and peoples’. This, in turn, is one reason why Dicey was ‘never entirely clear about [that] constitution’s territorial bounds’.^38^ The question did not matter: the same unitary principles supposedly applied throughout, but subject to the variety of different constitutional structures embodied in those general principles.

Dicey’s answer to the ‘how many constitutional orders’ question is not dual, like Bryce’s. It is at once unitary and plural—‘one’ and ‘lots’. But there is something unsatisfying about the unresolved tension between this ‘unitary constitution’ and the many different constitutional relationships concealed therein. The principles that embody that constitutional order—sovereignty, convention and the rule of law—are overwhelmingly general in nature and are neither intended nor able to direct the intimate operations of empire.

This suggests that Bryce’s account is more convincing than Dicey’s: that separate constitutional regimes prevailed within and beyond the pale. Perhaps, however, we can improve on both of these accounts. Bryce’s simple, binary division between empire and metropole understates the variety of relationships between the Crown and its various colonies. The British Empire was a network of possessions, dominions, protectorates, mandates, territories, ceded colonies, conquered colonies and, of course, the ‘home’ nations. There were further categories within these categories. The unique needs of separate territories and communities across the empire were served by a cluster of asymmetrical relationships. But there is a further problem with this dual model. A distinctive feature of the UK metropole—unlike the metropole in ancient Rome—is that it is interpenetrated by empire. The home nations include a settler colony, and this has constitutional consequences. Can we identify a metropole in this imperial constitutional order? If so, where does it stop and where does the empire begin? I will return to this point in section 3.

### B. Sovereignty

Dicey refers to the empire as a federation. He also describes Parliament having renounced its right to tax colonies. Students with a basic knowledge of Dicey’s thinking will realise that neither of these things should be possible in his scheme. So what did he mean, and what does it tell us about federalism and sovereignty?

Dicey claimed that, since the abolition of the Corn Laws, ‘no Imperial Act had been passed for the taxation of any colony’.^39^ He describes the omission to tax as the ‘renunciation’ of ‘the right’ to legislate by the imperial Parliament. Elsewhere, he describes this as a rule of ‘constitutional morality’.^40^ This is a striking claim. Dicey cannot mean Parliament has *legally* renounced its sovereignty. He says, in terms, that the

Imperial Parliament still claims in 1914, as it claimed in 1884, the possession of absolute sovereignty throughout every part of the British Empire; and this claim, which certainly extends to every Dominion, would be admitted as sound legal doctrine by any court throughout the Empire.^41^

Lino concludes that Dicey is simply dismissing the ‘*political* prospects for such a recurrence of imperial folly’.^42^ He repeatedly underlines the fact that Parliament was *legally* competent to change colonial legislation, and to tax the colonies.^43^ He concludes that ‘Dicey did not have a good answer for how the risk of imperial misrule in the dependent Empire, as against the settler colonies, was or could be constitutionally reigned in’. But Dicey did have an answer to this question. The central argument in Dicey’s constitutional theory is that an ‘absolutely sovereign legislature … should yet be bound by unalterable laws’.^44^ The originality of that theory lies in the synthesis of legal and political rules. The conventions of the constitution ‘have all one ultimate object … to secure that Parliament … shall in the long run give effect to the will of that power which in modern England is the true political sovereign of the State … the Nation’.^45^

Diceyan sovereignty is not a unitary, pure, absolute legal sovereignty. It is a compound sovereignty with legal and political dimensions. In Dicey’s eyes, the monarch in Parliament is the legal sovereign, but legal sovereignty is exercised subject to the supervisory sovereignty of the political sovereign.^46^ The body ‘the will of which is ultimately obeyed by the citizens of the state’ is ‘politically sovereign or supreme in a state’.^47^ Dicey identifies ‘the electors of Great Britain’ as ‘the body in which sovereign power is vested’.^48^ He later refers to ‘the will of the nation as expressed through Parliament’.^49^ At best, Dicey is advocating the sovereignty of an electorate of a few million men over an empire of half a billion people. At worst, he is advocating the sovereignty of a mythical race.

Dicey believes it is unconstitutional, and perhaps unthinkable, that Parliament would tax or otherwise interfere in the affairs of a fully responsible colony. Nonetheless, paradoxically, it is important that Parliament retains the notional legal power to do exactly this. In *England’s Case Against Home Rule*, Dicey says Parliament ‘cannot impose any legal limit to the exercise of its own power’. Nonetheless it may ‘excite expectations which it will be extremely difficult or hazardous to disappoint, and so may find itself morally fettered as to its subsequent legislative action’.^50^ He claims that

no constitutional lawyer will contend that the Parliament of the United Kingdom is legally bound by [the Declaratory Act of 1778]. But [the Act] makes it morally impossible for Parliament to tax any colony … The so-called Act establishes not a rule of law, but a precept of constitutional morality. It does not theoretically limit, but it practically impedes and interferes with the legislative sovereignty of Parliament.^51^

Dicey says the veto that both Parliament and government hold over colonial legislation helps to hold together the ‘the federation known as the British Empire’. The reference to the empire as a ‘federation’ is puzzling. Dicey was, of course, a stark critic of a federal empire because he thought federalism meant weak government. If the UK were to adopt an American-style federalism, then ‘the sovereignty of the Imperial Parliament would be destroyed and all English constitutional arrangements would be dislocated’.^52^ But, at the turn of the 20th century, ‘federalism’ did not only refer to the sort of ‘rigid’ juridical federalism that Dicey rejected. It could be used to refer to various sorts of political pluralism embracing a wide variety of structures of subsidiarity, including churches, political associations, trade unions and, indeed, empires.^53^ Dicey refers to the British Empire as a ‘federation’ because he thinks it can be a community of independent territories, under the umbrella of sovereignty and the Crown. To understand what he means, as Signe Rehling Larsen notes, ‘we need to retrieve the lost meaning of the federation as a discrete form of political association’.^54^ Dicey is referring to the ‘singular … complicated … anomalous … position of combined independence and subordination occupied by the large number of self-governing colonies … scattered throughout the British Empire’.^55^ The thing that holds together Dicey’s federation is the combination of political independence and legal subordination.

Parliamentary sovereignty grants Parliament a unitary legal authority over the UK. The trouble is, it grants Parliament unitary legal authority everywhere else too. Famously, as Ivor Jennings observed, it enables Parliament to ban smoking on the streets of Paris.^56^ But when we locate the example in France we miss the point. The point is that Parliament is legally competent to ban smoking in Dublin, Douglas and the District of Columbia. The structure enables Parliament to hold the reins of imperial constitutions even after colonies have secured wide powers of self-government. To see this clearly, we need to say a little more about the legal process by which empire was dismantled.

***

UK orthodoxy is that the constitutional independence of former colonies operates through the legal grant of power by the UK Parliament. Newly independent states view the matter differently.^57^ Parliament now has almost^58^ no power to legislate for the Commonwealth constitutionally, though it retains that legal power. It retained and used its legal and constitutional power to legislate for the dominions until at least 1931,^59^ when it enacted the Statute of Westminster. The Statute achieved this outcome in three ways. First, it repealed the Colonial Laws Validity Act 1868, which gave UK courts the power of strong-form judicial review over dominion and colonial legislation. Second, it memorialised a constitutional convention, shaped by the 1929 and 1930 Commonwealth Conferences, that no law shall extend to a dominion ‘otherwise than at the request and with the consent of the dominion’. Third, section 4 of the Statute creates a legally binding interpretive presumption in a similar form. The structures mean the constitutional convention—the political rule—did the work, even though the legal rule only extended to those dominions who adopted it.^60^ Here again, the hinge between politics and law is crucial to the devolution of power.

The Statute does not mention, still less grant, sovereignty or independence. In a more political and less legal sense, dominions were ‘independent’ long before it was enacted. As Kerr LJ notes in *Indian Association of Alberta*, which I consider in detail below,

independence, or the degree of independence, is wholly irrelevant … because it is clear that right and obligations of the Crown will arise exclusively in right or respect of any government outside the bounds of the United Kingdom as soon as it can be seen that there is an established government of the Crown in the overseas territory in question. In relation to Canada this had clearly happened by 1867.^61^

Dominions also remained substantially connected to the UK and to each other, not least through the indivisible Crown. The Preamble to the Statute attests to enduring allegiance to the Crown, and to the effect this has on succession to the throne. What this meant was that relations between dominions were subject not to rules of international law, but instead to imperial constitutional rules. So, for example, two dominions could not conclude a treaty, because the monarch who assumed obligations on their behalf was the same person.^62^ In turn, the suit of one colony by another colony ‘would be like the suit of the cook by the butler because too much was spent on coals’.^63^

Hamish Gray observed in 1960 that ‘the general tendency of constitutional lawyers is to reject the interpretation of [the Statute] which requires Parliament as a matter of law to act in a particular way for any particular purpose’. Indeed, less than five years after the Statute was enacted, Viscount Sankey restated what he thought was Diceyan orthodoxy:

[I]t is doubtless true that the power of the Imperial Parliament to pass on its own initiative any legislation that it thought fit extending to Canada remains in theory unimpaired; indeed the Imperial Parliament could, as a matter of abstract law, repeal or disregard section 4 of the Statute.^64^

In 1957, Parliament decided it was competent to repeal the Copyright Act 1911 not only at home, but also in its former dominions. The High Court of Australia did not share that view.^65^ But if we press this too far, we miss the wider point. Tom Nairn argues that in debates about home rule, ‘the crucial constitutional issue … [was] the conservation of the absolute sovereignty of the Crown in Westminster’. For Nairn, ideas such as these ‘are not bourgeois-constitutional trivia … they manifest the nature of the state and the whole material history which produced that state’.^66^ Legal sovereignty is not trivial, but neither is it central. In the imperial constitutional order, legal sovereignty was a distraction from the real location of political power. Questions of subsidiarity were settled not by reference to legal sovereignty, but by rules of ‘constitutional morality’.^67^ This ultimately accords with what Bryce had to say about flexible constitutions. Questions of subsidiarity might look like rigid, legal questions, but they are not. In an imperial scheme, they were supposed to be resolved by ‘customs, usages, understandings and beliefs bearing upon the methods of government’.^68^ Parliament retained a notional legal sovereignty over the empire—and everywhere else—but eventually the political sovereign had no more power in Dublin than they had in Paris.

This section considered two puzzles in the account of the UK constitution that emerged at the turn of the 20th century. The unitary constitution cannot mean ‘a constitution in which the same rules apply through the territory’, nor does it refer to a binary distinction between a metropole and an empire. It conceals multiple separate constitutional relationships. Moreover, the centralising sovereignty, characteristic of that theory, is a compound sovereignty with both legal and political dimensions. The central constitutional problem in this period—home rule—could not be adequately resolved with reference to legal sovereignty alone. It demanded a system of ‘constitutional morality’ which almost stayed the hand of the legal sovereign, and which protected the subsidiarity of dominions and colonies. Both of these ideas have important implications for the devolution settlement which currently prevails in the UK. I consider those implications in the next section.

## 3. A “coherent constitutional foundation”^69^

Shortly before the devolution settlement was enacted, Rodney Brazier said:

[E]ven after devolution the United Kingdom will still be a unitary state. The whole of that state, and all the parts that comprise it, will remain-at least ultimately under the jurisdiction of the Parliament of the United Kingdom and through it of Her Majesty’s Government in the United Kingdom.^70^

The previous section explained that the British Empire was characterised by multiple, overlapping constitutional orders under the *notional* legal superintendence of Crown and Parliament. It then explained that these structures are not supposed to achieve the subsidiarity every empire requires. The thing doing the work is political, not legal. Brazier was restating this orthodoxy, what David Feldman describes as ‘a constitutional vision dominated by the nineteenth century tradition of the United Kingdom as a unitary nation state and a Diceyan view of parliamentary sovereignty’.^71^ This section considers the conclusions of section 2 in a 21st-century context. It asks how many constitutional orders the UK has: none, one, two or several? Does the ‘jurisdiction’ of the Parliament of the UK, and ‘through it’ of the UK government, actually integrate those orders, as Brazier says?^72^

### ‘[T]he United Kingdom will still be a unitary state’

International law suggests a simple answer to the territorial question. The UK includes all territories for which the UK government is internationally responsible. The UK is responsible for devolved nations, the Crown Dependencies and the British Overseas Territories. If the government of Sark launched an armed attack on France, that would be an act of the UK. The UK is similarly responsible for a breach of the UK–EU–Norway Fisheries Agreement, regardless of whether the offending action takes place in Peterhead, St Helier or Stanley. Conversely, the UK is not responsible for foreign states. If Canada invaded the United States, this would not be an act of the UK. The Permanent Court of International Arbitration explained the point succinctly a century ago: ‘a State cannot adduce as against another State its own Constitution with a view to evading obligations incumbent upon it under international law or treaties in force’.^73^ No complex question of attribution or statehood applies.

Imperial constitutional theory suggests a more complex answer. Fifty-six member states acknowledge the king of the UK as the Head of the Commonwealth. Fourteen realms, including Canada, New Zealand and Papua New Guinea, share a head of state.^74^ Thirty-four territories, including independent states such as Jamaica, Mauritius and Brunei, allow appeals to the Judicial Committee of the Privy Council. Section 2(1) of the Ireland Act 1949 declares that Ireland ‘is not a foreign country for the purposes of any law in force in any part of the United Kingdom or in any colony, protectorate or United Kingdom trust territory’. *Halsbury’s Laws* reminds us that ‘New Zealand is for many if not all purposes a foreign state’, and in *Attorney General of New Zealand v Ortiz*, Lord Denning begged forgiveness for describing it as such.^75^ In Australia, in contrast, ‘the United Kingdom is for many if not all purposes a foreign country’.^76^ This makes it harder to draw a sharp constitutional distinction between the UK and states with separate international legal personality.

Jersey, Gibraltar or Jamaica might or might not be part of the UK, depending on the context. The Kilbrandon Commission said Overseas Territories and Crown Dependencies ‘in some respects … are like miniature states with wide powers of self-government’. Until 1950, the Crown Dependencies—unlike the British Empire—were regarded as part of the ‘metropolitan territory’ of the UK.^77^ Today, they are sometimes considered to be at home and sometimes considered to be abroad. The Interpretation Act 1978 defines ‘the United Kingdom’ as Great Britain and Northern Ireland.^78^ The Crown Dependencies plus the UK are ‘the British Islands’. Some statutory regimes apply in the UK and some throughout the British Islands. For example, the warranty regimes in the Investigatory Powers Act 2016 apply to the British Islands.^79^ In turn, *Halsbury’s Laws* tells us that British Overseas Territories are ‘not parts of the United Kingdom’ and that ‘their governments and law are distinct from [those of] the United Kingdom’.^80^

Sometimes the legal answer reflects the formal constitutional position and sometimes it draws on statute or custom. Sometimes it simply turns on whether people thought the territory was part of the UK, regardless of the formal position.^81^ In a contract, the words ‘United Kingdom’ sometimes follow the approach of the Interpretation Act^82^ and sometimes they do not.^83^ The use of the words ‘United Kingdom’ in a will or deed can include the Channel Islands.^84^ Sometimes we find all three approaches in the same judgment. In *Navigators and General Insurance Co Ltd v Ringrose*,^85^ a dispute on the application of an insurance contract, we see all three. Pearce LJ started his judgment with the constitutional position and the ‘guidance’ in the Interpretation Act 1889. He then considered custom and held that the term ‘United Kingdom’ did not include the Channel Islands. But he then concluded that the expression ‘within the United Kingdom’ covers ‘the area over which Her Majesty claims jurisdiction’.^86^ (As the Court of Appeal observed in *Partington*, His Majesty *does* claim jurisdiction over the Channel Islands.^87^)

Politically, an empire is one country. Legally, it can be lots of countries. When writing about domicile, Dicey invited us to think in political and legal terms, but also in geographical terms, historical terms and so on.^88^ As Frost and Murray note, ‘distinctions between “political” and “legal” aspects of the imperial government’s relationships with imperial territories played a prominent part in 20th-century efforts to insulate the British Empire from the developing international law of trusteeship and decolonisation’.^89^ In turn, as Roxana Banu argues, one consequence for the territorial story of the UK is that a great deal of work is still done by courts, applying rules of private international law.^90^ This partly explains the ambivalence described in the previous paragraph.

Imperial constitutional theory made sense of this diversity, organising its territorial constitution in three tiers, with the UK at the top, then below it the dominions, then below them colonies who had not obtained responsible self-government. There are problems at each tier. First, as we have seen, and will see, the UK includes a settler colony and, as we will see, some people thought of it as a Dominion. Second, as Donal Coffey writes, ‘it wasn’t exactly clear what set of characteristics defined a Dominion’, so ‘it could quite rightly be argued that the Dominion of Newfoundland was closer in political structure and outlook to that of Rhodesia than, say, Canada’.^91^ Third, at the bottom tier, the Crown’s powers varied considerably depending on whether the colony was ceded or conquered,^92^ not to mention the complex international arrangements which underpinned mandates and protectorates. At the heart of the picture is the old imperial idea of political tutelage. Territories are supposed to shift through these orbits as they gain autonomy. As they develop local institutions of self-government they grow more independent and shift further away from London. But this idea of ‘political tutelage’ was always an overbearing fiction. As Coffey notes, territories often ‘regressed to direct rule.’ They still do.

Fifty years ago, the Kilbrandon Commission reframed the three-tier model of government.. The UK was governed from Westminster and Whitehall, while Crown Dependencies and British Overseas Territories were governed locally. It described the Crown Dependencies—the Channel Islands and the Isle of Man—as ‘Unique miniature states with wide powers of self-government’ that were ‘not capable of description by any of the usual categories of political science … full of anomalies, peculiarities and anachronisms, which even those who work the system find it hard to define precisely’.^93^ In turn, the manual on overseas territory law describes this as ‘all that remain of the former British Empire’.^94^ Schedule 1 of the British Nationality Act 1981 identifies 14 self-governing British Overseas Territories, including Bermuda, the Falkland Islands and Gibraltar.

But as we will see, the powers that characterise each relationship with the centre vary significantly. The imperial territorial constitution was not a solar system so much as a nebula, and the UK’s current territorial constitution exists not in fixed tiers, but in loose bands. Westminster and Whitehall form the innermost circle. Wales, Northern Ireland and Scotland are somewhere in the middle. Further out is a belt containing the Crown Dependencies and British Overseas Territories, which are in some senses less dependent and in some sense more. At the farthest frontier of the UK constitution order lie an outer band of states, including the Commonwealth. These are self-governing states, but subject to important anomalies, such as the enduring jurisdiction of the Privy Council.

What is at the centre of this picture? Is it the UK? Is it Great Britain? Is it England? Anthony King famously left Northern Ireland out of *The British Constitution*, both in substance and in title. He said, ‘What happens in Northern Ireland scarcely affects British constitutional development; constitutional development in Britain scarcely affects what happens in Northern Ireland’.^95^ King’s move is to separate core and periphery. The core—the metropole—is the place where the normal rules apply; the periphery is the exception. We might be inclined to say, against King, that an account of the UK that excises Northern Ireland is an inadequate account—it simply does not identify the thing people mean when they talk about the UK. The UK constitution ought to describe all of the territory, not just the salient parts. But if we make this move, where *do* we draw that line? Should courses in Public Law begin in Alderney?

The obvious response is ‘they should begin in London.’ But this opens up a new set of problems. Around the time of the devolution settlement, David Feldman described ‘a distinctive view of the constitution in Scotland’ according to which ‘there could be no assumption that the [UK] Parliament would have the same characteristics and competencies as either the former Scottish Parliament or the former English Parliament’, citing Lord President Cooper in *McCormick* and Lord Keith in *Gibson*.^96^ Feldman claims English constitutionalists ‘so far as they have thought about the matter at all have tended to assume that English institutions continued unchanged’. In *Lord Gray’s Motion*, Lord Hope did not express an opinion, but stated that the argument that parliamentary sovereignty was fettered by the Treaty of Union ‘cannot be dismissed as entirely fanciful’.^97^ The ‘distinctive view of the constitution in Scotland’ is a pluralist view. It argues that there are at least two—and probably many—constitutional orders in force on the territory of the UK. This view surfaces after the second world war, but before the British Empire sunders.

It is worth underlining that this is a Unionist view. It is presented as an account of Union, not as a reason to reject Union. In contrast, Scottish Nationalists such as Ronald Murray and Neil MacCormick urge a monist answer to the question. They argue that the metropole is clearly England: this is why most accounts of the UK constitution start there.^98^ They argue that ‘the extensions of the sway of the British State, however important geopolitically, were byways from the point of view of the evolution of an essentially unitary constitution’.^99^ MacCormick’s argument is that the UK is an incorporating union, one in which England drew other nations into its orbit. That union had a single, coherent unitary constitutional order, and it was an English order. This is an intentionally unattractive account of the UK constitution. It is designed to expose the unequal terms on which the UK became a unitary state, and to press for more radical constitutional change to rectify that inequality. It is ironic that the recent approach of the Supreme Court has tacked much closer to Neil MacCormick’s vision of the Union than to that of Thomas Cooper.^100^

***

In a recent article, Martin Loughlin asks a similar question to Rodney Brazier: is the UK ‘sufficiently integrated with respect to territory, communities, and ruling authority to be capable of possessing a coherent constitutional foundation’?^101^ The answer he gives is ‘no’.

Loughlin’s focus is Ireland. He argues compellingly that the constitutional nature of Ireland has always been imperial, and that Northern Ireland’s governing institutions should be compared to ‘former settler colonies seeking Dominion status, such as Australia or Canada’.^102^ In 1931, the Statute of Westminster specified that the Irish Free State was itself a dominion. Loughlin characterises Northern Ireland before 1972 as ‘devolutionary in form, but Dominion in practice’.^103^

It is easy to agree with this central claim. The UK is not sufficiently integrated to possess a coherent constitutional foundation. Northern Ireland is *both* a settler colony and part of the UK. The metropole and the empire overlap. But the foil for Loughlin’s argument is the same four-cornered unitary state described in the introduction. His argument is that Ireland was—and is—the exception to a British rule, and that the UK would possess a coherent constitutional foundation but for that exception. It is hard, however, to identify the coherent constitutional order that can be contrasted with the Irish exception.

For one thing, we might ask whether Scotland was integrated into the British system. Loughlin notes that Ireland was exceptional because ‘the law courts remained in Dublin’.^104^ Scotland’s courts remained in Edinburgh, and they applied separate law. Loughlin also notes that Ireland was exceptional because the Northern Ireland civil service ‘is organised on a separate basis from the rest of the UK civil service’.^105^ But the Home Civil Service is not unitary: the Scottish Civil Service has been organised on a separate basis since before Partition.^106^ And unionists do not see Ireland as a dominion, connected to the mother country: they think they live in the mother country.

Where, then, do we look for the unifying vision of the UK constitution, if not in the courts or the government? The central argument, both in this piece and in Loughlin’s wider work, is that Parliament is the constitutional foundation, and that its legal authority and political coherence are the central characteristics of the UK constitutional order. ‘Parliament’s absolute authority to make law’ is ‘the fundamental principle of the British constitution’.^107^ Ireland supposedly stands apart from that, because it has a separate politics and separate system of government oriented around the constitutional question.^108^ Loughlin argues, repeatedly and uncontroversially, that ‘the British sought to rule Ireland without the consent of the great majority of its population’.^109^ But, of course, in this period^110^ the British ruled *Britain* without the consent of the great majority of its population.

The reason why the UK lacked a coherent constitutional foundation in this period is not because *Ireland* was different. Compare the United States, which has five imperial territories. Puerto Rico, the US Virgin Islands, American Samoa, Guam and Mariana are parts of the United States and their acts are attributable to Washington. But they are not states. Nonetheless, it might still be fair to characterise the United States as a federation. The territories are clear exceptions to a clear federal scheme: what happens in Guam scarcely affects federal constitutional development. In contrast, the nature of the UK “federation” makes it hard to identify the exception and the rule.

Loughlin, King and MacCormick are not describing the territory of the UK so much as theorising it. We can only decide which systems to include based on some account of the territorial constitution, and each theorist is proposing one. The underlying question is a problem for constitutional theory: why should we include Ireland but exclude the Isle of Man? Is the UK constitution an amalgam of the various constitutional rules that apply in the territory or is it a centralising structure, a synthesis or simplification of those rules? Is it the normal rules, the central rules or the rules of the metropole which take precedence, or is it a series of very general rules, qualified by exceptions? If, together with King, we think the UK is the place where the normal rules apply, then we need a satisfying account of the UK metropole. But, as we have discovered, it can be hard to say where the metropole lies because the UK is freighted with multiple overlapping constitutional orders.

If we draw back for a moment, MacCormick, Loughlin and King arrive at the same destination but by different routes. They assert that there could be a coherent foundation to the territorial constitution. But that constitution is not the set of the constitutional rules that apply in the territory of the UK; rather, it is purer set of rules that apply at the heart of the territory, a notional English constitution. Both metropole and empire were, and are, more complicated than this. Ireland is singular, but so too are Scotland, Jersey and Gibraltar. Even if we just focus on dominions for a moment, they did not have singular governance arrangements. Our current arrangements braid together some of the central features of the imperial order with aspects of dominion governance, along with more recent innovations. Any satisfying theory of the constitution must account for this diversity, not simplify it away.

### ‘[U]nder the jurisdiction of the Parliament of the United Kingdom’

The UK never wrote a constitution for itself.^111^ It wrote them for others.^112^ The imperial constitution was flexible, but colonial institutions worked under rigid legal constitutions. Besides the UK, only two former possessions (New Zealand and Israel) do not have a written constitution. Ninety further British possessions are subject to written constitutions, not to mention the many substate constitutions formerly supervised by London.^113^

These constitutions were originally superintended by Parliament. As late as 1982, Canada had to request an Act of the UK Parliament to ‘patriate’ its constitution—to deprive the UK of the legal power to change the Canadian constitution. Section 2 of the Canada Act 1982 provides that ‘no Act of the Parliament of the United Kingdom passed after the Constitution Act, 1982 comes into force shall extend to Canada as part of its law’. In light of the argument about the Statute set out above, we might ask why they bothered. Legal sovereignty is not a solution to problems of subsidiarity, and indeed it cannot be. It turns boundary problems, problems to do with the overlap of competence, into problems of ‘constitutional morality’. It turns questions of law into questions of politics.

One reason why the Parliament needed this limitless sovereignty was because it held the reins of limited parliaments across the empire. In this structure, the power of the imperial Parliament was constituent, while the power of the dominions was constituted. Dicey describes Parliament in this constituent role as the ‘ever-wakeful legislator’.^114^ We might, in turn, imagine this imperial parliamentary sovereignty as the operating system that ran the constitutional software of empire. It allowed updates and patches to be issued, when needed. It also enabled remote access when London thought that expedient. Limited government under central supervision was clearly thought to be desirable. Meanwhile, the metropole nobly forewent these benefits, the better to secure them for others.

The outcome is that the UK is left with the software that reboots other people’s constitutions. While former colonies run their own operating system, the UK stares at a command prompt.

UK territories, present and former, are subject to a model of subsidiarity maintained not by law, but by ‘constitutional morality’. As a matter of UK law, the UK Parliament is *legally* competent to legislate for Crown Dependencies and Overseas Territories regardless of how wide their powers of self-government are supposed to be.^115^ The superintendence of the UK Parliament extends to Sark and Stanley as much as it does to Stormont.^116^ But *constitutionally* (as distinct from *legally*), Parliament has almost no power to legislate for the Commonwealth. It also has a *constitutionally* limited power to legislate for Crown Dependencies and devolved nations.^117^ In turn, the UK Parliament has a constitutionally *unlimited* power to legislate for Overseas Territories. For example, the Sanctions and Anti-Money Laundering Act 2018 (SAMLA) required the government to legislate to ensure that Overseas Territories maintain Registers of Beneficial Ownership.^118^ The government retains the power to make primary legislation for Overseas Territories by Order in Council.

Parliament has a constitutionally limited, but legally unlimited, power to legislate for Crown Dependencies and devolved nations.^119^ There is a convention that limits Parliament’s legislative powers over Crown Dependencies to the fields of defence, nationality, citizenship, Succession to the Throne, extradition and broadcasting.^120^ By the same convention, it ‘almost invariably’ legislates with the consent of the Crown Dependency. But, like the words ‘not normally’ in the Sewel Convention, the words ‘almost invariably’ can be taken to mean ‘sometimes’. For example, the Fisheries Act 2020 included a clause enabling the UK government to apply the Act to Crown Dependencies by Order in Council. It did so ‘despite explicit opposition from Jersey and Guernsey’.^121^

The thing doing the work is not legal sovereignty, but a looser and more political rule. Section 2 argued that the supremacy of Parliament is a standing legal exception to any rule that supposedly distinguishes devolved and reserved powers. In 1968, the UK Parliament sought to legislate for Rhodesia, a self-governing colony which had recently issued a unilateral declaration of independence. In *Madzimbamuto v Lardner Burke*, the judicial committee of the Privy Council said

it is often said that it would be unconstitutional for the United Kingdom Parliament to do certain things … But that does not mean that it is beyond the power of Parliament to do such things. If Parliament chose to do any of them the courts could not hold the Act of Parliament invalid.^122^

This is another example of a phenomenon we explored in section 2. It turns a problem of legal sovereignty into a problem of political sovereignty. The judicial committee also said:

If The Queen in the Parliament of the United Kingdom was Sovereign in Southern Rhodesia in 1965, there can be no doubt that the [Act was] of full legal effect there. [I]n 1923, full Sovereignty over the annexed territory of Southern Rhodesia was acquired. That Sovereignty was not diminished by the limited grant of self-government which was then made …^123^

The reference to sovereignty is unorthodox. Presumably, on this account, Parliament was not sovereign in Paris in 1965 and therefore could not ban smoking. The Committee concluded that there was ‘no intention to transfer Sovereignty’. But it added ‘or any such clear-cut division between what is granted by way of Sovereignty and what is reserved’. The latter formula is necessary because, as we have seen, the thing granted to the dominions was not sovereignty, but something short of sovereignty. Here, the work is being done by a principle of statutory interpretation. Parliament is presumed not to legislate extraterritorially. But, of course, Parliament can (and does) legislate extraterritorially. Even if the Queen in Parliament were not ‘fully sovereign’, it might still be hard to read down the intent that ‘The Southern Rhodesia Act’ should apply in South Rhodesia.

These imperial structures survive. In 2010, in *Barclay* the Supreme Court concluded that Parliament did not intend for English courts to entertain a challenge to the compatibility of Sark legislation with the European Convention. Drawing on *Madzimbamuto*, it said

in the eyes of the courts the UK Parliament did have a paramount power to legislate for the Islands on any matter, domestic or international, without their consent, although it should be no more ready than in the past to interfere in their domestic affairs…^124^

The superintendence of the UK Parliament extends to Sark as much as it does to colonies or devolved nations, in spite of any constitutional differences between those territories.

Recall Loughlin’s objection that ‘the British sought to rule Ireland without the consent of the great majority of its population’. All UK territories share a central Parliament, but not all residents of UK territories are represented in that Parliament. Northern Ireland is within the pale. The Isle of Man, however, is not, because Crown Dependencies and British Overseas Territories have their own representative institutions. The trouble, nowadays, is that Scotland, Wales and Northern Ireland also have their own representative institutions.

Representation in Westminster has not always been confined to the UK.^125^ However, broadly speaking, James Bryce was right: the inhabitants of the UK are represented by the UK Parliament while the inhabitants of the British Empire are not. At that time, Parliament was the only representative institution for the home nations. In 1920, Ireland was granted two further Parliaments, over and above its representation at Westminster. There are now four legislatures in the UK and nine in the British Islands, some of which overlap with Westminster and some of which do not. It is hard to explain why Scots should elect representatives to both Holyrood and Westminster, while Manx people are only represented in the Tynwald and the English only at Westminster. No significant legal obstacle lies in the way. The Admissions Clause of the US Constitution prevents Congress admitting Puerto Rico to the Union without the consent of state legislatures. The UK could achieve that result by simply amending the Parliamentary Constituencies Act 1986.

A constitution by which a small metropole governs territories to which it is not electorally accountable is not a good constitution. Perhaps people tolerate this state of affairs because it is how things have always been done. The advent of devolution makes it difficult to have one rule for the dependencies and territories and another for home nations. If Crown Dependencies and Overseas Territories are abroad, if they are functionally independent territories with their own representative institutions, then we can easily explain why they are not represented at Westminster. But if the mode of independence they enjoy looks more like Scotland or Wales, then it is hard to explain why they should be different.

### A. ‘[A]nd through it of Her Majesty's Government in the United Kingdom’

The UK constitution makes genuine federalism impossible. However, its sovereignty is supposed to be tempered by a deeper federalism, a constitutional morality which preserves the subsidiarity of ‘federation’. Perhaps that morality holds ‘almost invariably’. Perhaps it holds ‘normally’, perhaps less.

Robert Blackburn wrote in *Halsbury’s* that ‘The United Kingdom and its dependent territories within His Majesty’s dominions form one realm having one undivided Crown’.^126^ In the same vein, John Finnis argued that:

we need not flinch to say that this one undivided realm has an imperial character, and that the acts of Her Majesty in giving instructions to … colonial governors are acts of the imperial Crown. [The] power is of course exercised on the advice of UK ministers … and is exercisable, of course, not only in the interests of good government of the colony in question but also in the interests of trade within the empire as a whole, or the defence of the whole realm, or the international obligations of the United Kingdom, and so forth.^127^

Neil MacCormick once said what ‘elsewhere is treated as a unitary acting state is in the United Kingdom discussed through doctrines about “the Crown”’. Maitland described the Crown as a ‘convenient cover for ignorance: it saves us from asking difficult questions’.^128^ The questions the Crown saves us from asking include ‘who governs?’, ‘where?’, ‘in whose interests?’ and ‘according to which rules?’

The Crown is a vast and subtle topic, and it is not possible to encapsulate its majesty here. Neither can we ignore it in an article on this topic. This section makes a point about the Crown and the public interest, and its importance for the territorial constitution. If the realm and Crown are indivisible, this leaves little space for territorially separate conceptions of the public interest. The move is similar to the section above. Like sovereignty, the Crown represents a supervening, central public interest. Like sovereignty, while it notionally respects local interests, it in fact enables the centre to pre-empt them at any time, no matter how fundamental they are. This idea is not conducive to a legal scheme of subsidiarity of the sort contemplated by the 1998 devolution settlement. It is a clear example of the difference between imperial and federal public law.

All UK territories share central government. The Justice Committee of the House of Commons recently observed that the responsibility for government is ‘shared between the dependencies and the UK’. In respect of the Dependencies, the Crown retains the power to legislate by Order in Council.^129^ In turn, legislation by the parliaments of the Dependencies requires royal assent, the award of which is not a formality. The UK Ministry of Justice ‘examines each piece of legislation to ensure there is no conflict with the UK’s international obligations (including the European Convention on Human Rights) or with any “fundamental constitutional principles”’.^130^ A 2013 inquiry by the Commons Justice Committee^131^ found that UK civil servants also sought to harmonise the content of insular legislation with ‘UK policy’.^132^

The relationship between the Crown and British Overseas Territories is even simpler: Whitehall can govern them directly. Once again, this is an imperial model: even in the case of the dominions, direct rule could be imposed in certain cases.^133^ The Turks and Caicos Islands were governed by direct rule between 2009 and 2012. In April 2022, a Commission of Inquiry recommended that self-government in the British Virgin Islands should be dissolved and the territory should be run directly from London. The reason was a major corruption scandal in the territory,^134^ though in the same period, a major corruption scandal led to the collapse of the UK government.^135^ In Northern Ireland, the work of the Assembly and the Executive has been suspended by the UK government six times since the Assembly’s establishment in 1998.

Many people might settle for a model of subsidiarity in which the UK government respected a devolution settlement ‘almost invariably’ rather than just ‘normally’. But all of these words mask a large—and largely discretionary—measure of central government control over all British territories, embodied in the Crown. The wider point, which I have explored above, is the lack of any clear distinction between home and abroad save for ‘wide powers of self-government’. The closest we might get is a focal model, where powerful territories drift towards the outer orbits and less powerful ones are drawn to the inner ones. This model draws on the imperial predecessors described at the beginning of section 3.

The UK is sometimes described as a voluntary union of nations.^136^ One reason for this is because the Commonwealth modelled itself as a voluntary association of free and independent states.^137^ The Balfour formula imagined the Commonwealth as an association of

autonomous communities within the British Empire, equal in status, in no way subordinate to another in any respect of their domestic or internal affairs, though united by a common allegiance to the Crown, and freely associated as members of the British Commonwealth of Nations.^138^

Vernon Bogdanor argues that the dominions ‘came to be linked together not through being under the legislative supremacy of a single Imperial Parliament … but through allegiance to the Crown’.^139^

One practical effect is that when London-based officials make decisions about UK territory they are not always acting as ‘British’ officials.^140^ In the 18th and 19th centuries, the Crown was theoretically indivisible, and the UK and its empire formed a single realm. Contrary to Finnis’s view above, Donal Coffey explores the demise of this idea between the abdication crisis and the Indian Partition.^141^ It was not possible to maintain the theory of an indivisible Crown where the units made separate decisions according to separate conceptions of the public interest.

In the 1980s, the Indian Association of Alberta sought to enforce Crown obligations against the government of the UK. The basic question was whether the UK was constitutionally responsible for Canada. The Court of Appeal rejected the claim. The crown was ‘to be treated as divided’. Previous proclamations, promises and treaties were no ‘longer binding on the Crown in respect of the United Kingdom’.^142^ Kerr LJ made a separate, fiscal argument that ‘the liabilities of the Crown in right of … one of the Crown's territories can be satisfied only out of the revenues, and by the authority of the legislature, of that territory’.^143^ This comes close to a theory of subsidiarity: territories are independent to the extent that they can independently mobilise resources, digest them and turn them into social goods.^144^

Twenty years later, in *Quark Fishing*,^145^ the House of Lords entertained a similar challenge to a government decision under section 6 of the Human Rights Act. The Secretary of State argued that they were acting on behalf of the Crown of South Georgia, not the Crown of the UK, so they were not a public authority for the purposes of the Act. Following *Indian Association of Alberta*, Lord Bingham held that the Crown was divisible, into what he described as separate ‘capacities’, each denoting a ‘system of government within which the exercise of executive power takes place’.^146^ But the governor of South Georgia acts on the instruction of the Foreign Secretary. Hence, capacity in this context cannot refer to a separate executive government. Rather, it refers to the separate conception of the public interest that the decision maker is supposed to promote.

In *Bancoult*, the Crown decided to exile the population of the Chagos Islands by Order in Council.^147^ The Divisional Court and the Court of Appeal thought the power to issue the Order was limited to action for the ‘peace, order and good government’ of the territory and exile was not of that character. The majority of the House of Lords disagreed. Lord Hoffmann said that ‘Her Majesty in Council is … entitled to legislate for a colony in the interests of the United Kingdom’, and in the event of a conflict of interest, ‘she is entitled, on the advice of Her United Kingdom ministers, to prefer the interests of the United Kingdom’.^148^ Such a minister would act ‘in the interests of the undivided realm’.

The Crown was united prior to the abdication crisis. It was divided in *Indian Association of Alberta*, then reunited by the time *Quark Fishing* was decided. Cases such as these turn on whose interests are to be taken into account, and by whom, when a decision is made. The point of dividing the Crown is to distinguish between the interests of Canada, South Georgia or the British Indian Ocean Territory and the wider interests of the UK. It appears that the Crown divides when those interests need to be divisible and unites when they need to be indivisible. As Baroness Hale said in *Quark Fishing*, this approach is an ‘air of complete unreality’.^149^ The Crown is an abstraction. It is no basis on which to make sensible decisions about whose interests matter.

Anne Twomey argues that, when ministers of a particular territory acquire the right to render separate advice to the monarch, the Crown divides.^150^ She draws on Australian examples prior to the 1986 Australia Act. Until then, Australian provinces (unlike Canadian provinces) were separate from the Commonwealth of Australia, and each had a separate Crown. This fact had institutional consequences. Australian provincial ministers had the power to advise the monarch, while Canadian provincial ministers were appointed by and advised through the Governor General. Each ‘Crown’ represented a new dimension of the public interest.

On this basis, Twomey speculated that each devolved nation might have a separate Crown, reflecting a separate, territorial public interest.^151^ She asked

must it now be accepted that the Queen acts as Queen of Scotland, Queen of Wales, and Queen of Northern Ireland, and the Scotland Act 1998, the Government of Wales Act 2006 and the Northern Ireland Act 1998 are not part of the law of the United Kingdom because they confer constitutions on separate governments?

She also noted that, ‘in advising the Queen on Scottish matters … [British Ministers] could only take into account Scottish interests and could not take into account the interests of the United Kingdom as a whole’.^152^

The First Minister of Scotland is a Minister of Crown with separate access to the Palace. For most purposes, the doctrine of cabinet collective responsibility means it is not important to specify which minister tenders advice to the monarch. But this raises a theoretical problem: which minister gets to tender advice. According to Twomey’s scheme, the fact that Scotland gets to tender separate advice means it is a separate realm with a separate Crown.

This probably goes too far. The office of First Minister might better be characterised as a special statutory office, which does not work in the same conventional fashion as its imperial predecessors.^153^ In particular, the conditions under which it is held are substantially different to other ministers and there are Privy Council-level processes which manage the tendering of advice. Scotland is probably a special case—a pseudo-realm with a pseudo-Crown.

Drawing back, we can see two points of wider importance. First, Frederic Maitland and Baroness Hale are correct. When presented with a question such as ‘whose interests should we take into account?’, it is atavistic and surreal to reach for an imperial abstraction. What we need are structures which identify those interests. Second, when we built new devolutionary structures, we did so without considering what Britain actually was. The settlement ought to have acknowledged separate interests in separate parts of our territory, and built structures that reflected that. We now have a constitution whereby, in Whitehall, the separate interests of different parts of the UK are represented in at least five separate government ministries. Scotland, Northern Ireland and Wales have their own departments. The Ministry of Justice represents the interests of the Crown Dependencies. The interests of Overseas Territories are represented by the Foreign, Commonwealth and Development Office.^154^ As Lord Hope said, obscurely, in *Quark Fishing*, a decision about a UK territory might nevertheless ‘be concerned exclusively with … foreign policy’.^155^

The interests of England, of course, are wrongly assumed to be indistinguishable from the interests of the UK and are represented by the UK government without differentiation.

## 4. ‘[W]hat law had left undecided …’

We sometimes describe the UK as a voluntary union of nations.^156^ Perhaps the UK is best described as a union to which some parts originally consented, some parts continue to consent and some parts never consented. The UK is not a unitary state where all parts bear a singular relationship to the centre, nor is it a post-unitary federal state. If anything, ‘asymmetric devolution’ tends to understate its complexity, which cannot easily be reduced to three notional lines of symmetry. The UK is an imperial state, an archipelago, a nebula. Each part bears a singular relationship with London. It is a state characterised by multiple constitutions, laminated with the veneer of sovereignty and the Crown.

There is at least one move still available if we wish to maintain that the UK is a unitary state. We can concede the foregoing: the multiple constitutional orders; the facade of legal sovereignty; the variety of administrative relationships; the variable nature of the public interest; and in all of them the echoes of imperial structures we supposedly dismantled. We can then say ‘but this is simply what it means to be a unitary state’. UK territories are restricted by laws they cannot change. They are not federal, so they must be unitary. The answer to the question ‘how many constitutions?’ may as well be ‘one’.

This is an intellectually coherent move, but the price of that coherence is artificiality. Britain was not a unitary state in the same sense that Iceland is a unitary state. It was, at best, a special sort of unitary state. The legal definition of our territorial constitution does not describe the many constitutional orders present in our state. If the UK constitution is unitary, it is unitary only insofar as it *integrates* these constitutional orders. And if we wish to define our territorial constitution as the place where the ‘normal’ rules apply, then where is that place? If our answer to that question is ‘England’, then that is an unattractive answer. The incorporating union is not a redeeming theory. It is groundwork for the argument that the union should be dismantled.

The flexible constitution is intentionally and perhaps constructively ambiguous on these matters. It makes it hard to say who should be represented by whom, where and for what purpose. In this way, it creates two sorts of instability. It vests a legal power in the centre, to recall any grant of independence to any part of the empire. This is an understandable cause of concern for territories such as Guernsey, who suddenly find that Parliament can and will legislate without their consent. On the other hand, the imperial constitution emphasises the hollowness of that legal power. It makes subsidiarity subject to politics, not law. As Dicey belatedly said ‘force would no doubt settle what law had left undecided’.^157^

It is common to talk of devolution as a waypoint towards some notional end state. Unionists see it as a point on a journey to disunion. Tam Dalyell famously said devolution was a ‘motorway without exit to a destination … indistinguishable from a separate state.”^158^ In contrast, nationalists talk about devolution as a return ticket to greater central control. They see it as an eccentric form of power-sharing, in which only one side actually needs to share. The imperial constitution, based on a peremptory sovereignty, makes devolution provisional because it makes independence provisional. All sorts of self-government are in the gift of the Westminster Parliament, but the gift is always revocable.

The point, for both sides, is that our current arrangements are unstable because imperial constitutional structures amplify *both* centrifugal and centripetal forces This instability—this ‘flexibility’—is a feature of the imperial constitution lauded by Dicey and Bryce. As Dicey wrote, ‘in point of pliability, power of development, and freedom of action, English constitutionalism’ excels, and these qualities are ‘essential to the maintenance by England of the British Empire’.^159^ As Bryce wrote, these constitutional arrangements are conducive ‘to a State which is expanding its territory and taking in other communities’.^160^ Perhaps, in future, they will also be conducive to a state that needs to contract its territory.

## Footnotes

1 NW Barber, *The United Kingdom Constitution: An Introduction* (Clarendon Press 2022) 217: ‘The UK is often presented as the paradigm of a unitary state’; Anthony Bradley, Keith Ewing and Christopher Knight, *Constitutional Law* (Pearson 2022) 32: ‘the United Kingdom is often described as a unitary state since there is no structure of federalism’.

2 Roger Masterman and Colin Murray, *Constitutional and Administrative Law* (CUP 2022) 437: ‘Prior to devolution the UK was clearly a unitary state’.

3 David Edgerton, *The Rise and Fall of the British Nation* (Penguin 2019) 28.

4 CF Strong, *Modern Political Constitutions* (Macmillan 1972) ch 4, s IV.

5 See Barber (n 1) Introduction; cf Vernon Bogdanor, *The New British Constitution* (Hart Publishing 2009).

6 Statute of Westminster 1931.

7 Rhodesia and South West Africa were in a state of transition towards self-government.

8 Declaration of Perth, 18 May 1968.

9 Canada Act 1982; Australia Act 1986.

10 (Cm 3658, 1997).

11 See 2025 Treaty on the British Indian Ocean Territory/Chagos Archipelago (UK–Mauritius).

12 Tom Nairn, *The Break-Up of Britain* (Verso 2021).

13 The word refers to the homeland, parent state or central territory of an empire.

14
*R (Quark Fishing Ltd) v Secretary of State for Foreign and Commonwealth Affairs* [2005] UKHL 57, [2006] 1 AC 529.

15 Neil MacCormick, *Questioning Sovereignty: Law, State, and Nation in the European Commonwealth* (OUP 1999) 28.

16 Stephen Howe, *Empire: A Very Short Introduction* (OUP 2002) 14. See also the theoretical discussion in Benjamin Cohen, *The Question of Imperialism: The Political Economy of Dominance and Dependence* (Basic Books 1973).

17 ibid 15.

18 Royal Commission on the Constitution, *Report of the Commission* (Cmnd 5460, 1973) 543 (Kilbrandon Commission).

19 Anthony Bradley and Keith Ewing, *Constitutional and Administrative Law* (Pearson 2018) 39.

20 Barber (n 1) 1, 18.

21 Signe Rehling Larsen, *The Constitutional Theory of the Federation and the European Union* (OUP 2021) 13. Note that Larsen uses this definition to connect ‘fédération, Bund, confederation, union of states, and federal union’.

22 Kenneth Wheare, *Federal Government* (OUP 1964) 33.

23 There are other ways to conceive of the unity of the state, but I cannot pursue the point here.

24 Paul Craig, *Public Law and Democracy in the United Kingdom and the United States of America* (Clarendon Press 1990).

25 James Mitchell, *Devolution in the UK* (Manchester UP 2009).

26 WJM Mackenzie and Bill Chapman, ‘Federalism and Regionalism’ (1951) 14(2) MLR 182.

27 See eg Neil Walker, ‘Beyond the Unitary Conception of the United Kingdom Constitution?’ [2000] PL 384, 395.

28 Paolo Carozza, ‘The Problematic Applicability of Subsidiarity to International Law and Institutions’ (2016) 61(1) Am J Juris 51. See also A Follesdal, ‘The Principle of Subsidiarity as a Constitutional Principle in International Law’ (2013) 2(1) Global Constitutionalism 37; NW Barber, ‘The Limited Modesty of Subsidiarity’ (2005) 11(3) ELJ 308.

29 James Bryce, *Studies in History and Jurisprudence* (OUP 1901) 132.

30 ibid 164.

31 ibid 141.

32 ibid 133–4.

33 Dylan Lino, ‘Albert Venn Dicey and the Constitutional Theory of Empire’ (2016) 26(4) OJLS 751; Dylan Lino, ‘The Rule of Law and the Rule of Empire: A.V. Dicey in Imperial Context’ (2018) 81(5) MLR 739.

34 D Feldman ‘None, One or Several’ (2005) 64(2) CLJ 329.

35 Josep Maria Fradera, *The Imperial Nation: Citizens and Subjects in the British, French, Spanish, and American Empires* (Princeton UP 2018), cited in Eva Nanopoulos, ‘The Material Constitution and Imperialism’ in Marco Goldoni and Michael Wilkinson (eds), *The Cambridge Handbook on the Material Constitution* (CUP 2023). See also Michael Hechter, *Internal Colonialism: the Celtic Fringe in British National Development* (Routledge 1999).

36 AV Dicey, *Introduction to the Study of the Law of the Constitution* (Macmillan 1915). This is further explored in Lino, ‘Rule of Law and the Rule of Empire’ (n 32). See also the discussion of the Coercion Acts in AV Dicey, *England’s Case Against Home Rule* (John Murray 1886) ch 3.

37 Herbert Kaufman, *The Forest Ranger: A Study in Administrative Behavior* (Johns Hopkins Press 2006).

38 Lino, ‘Albert Venn Dicey’ (n 33) 759.

39 Dicey, *Introduction* (n 36) Introduction fn 24.

40 Dicey, *England’s Case Against Home Rule* (n 36) 246.

41 Dicey, *Introduction* (n 36) xliii–xliv.

42 Lino, ‘Rule of Law and the Rule of Empire’ (n 33) (original emphasis).

43 ibid 774.

44 AV Dicey, *Thoughts on the Union Between England and Scotland* (Macmillan 1920) 252.

45 ibid 290–1.

46 See also Bryce (n 29) 511 *et seq*.

47 Dicey makes a similar claim, Dicey, *Introduction* (n 36) cix, 27–8, 290–1.

48 ibid cix, 27–8, 290–1.

49 ibid 456. cf AV Dicey, ‘The Development of Administrative Law in England’ (1915) 31 LQR 148, 149.

50 Dicey, *England’s Case Against Home Rule* (n 36) 245.

51 ibid 246.

52 ibid 168. See also Barber (n 1) 1.

53 See David Runciman, *Pluralism and the Personality of the State* (CUP 1997) 71–2, 79–80, 150, 152, 177–8, 187.

54 Larsen (n 21) 14. She argues, contrary to Dicey, that the federation can be defined by the absence of one overarching sovereign.

55 Dicey, *England’s Case Against Home Rule* (n 36) 198.

56 Ivor Jennings, *The Law and the Constitution* (University of London Press 1959).

57 Consider, for example, the Irish and Indian constitutional responses to the purported grant of independence by Westminster. In art 295, the Indian Constitution 1950 formally abolished the Government of India Act 1935. cf art 2 of the 1922 Constitution of the Irish Free State; and the sustained attack on the Statute of Westminster by the Oireachtas between 1932 and adoption of the 1937 Constitution.

58 The limited exceptions to this rule are not important for our purposes. See eg the Succession to the Throne Act 2013.

59 Thomas Mohr, ‘British Imperial Statutes and Irish Sovereignty: Statutes Passed After the Creation of the Irish Free State’ (2011) 32(1) Journal of Legal History 61.

60 Australia, for example, only adopted the statute through the Statute of Westminster Adoption Act 1942.

61
*R (Indian Association of Alberta) v Secretary of State for Foreign and Commonwealth Affairs* [1982] QB 892, 927.

62 Arthur Berriedale Keith, *The Sovereignty of the British Dominions* (Macmillan 1929).

63 T Baty, ‘Sovereign Colonies’ (1921) 34(8) Harv L Rev 837, 846. I am grateful to Roxana Banu for the reference.

64
*British Coal Corporation v The King* [1935] AC 500.

65
*Copyright Owners Reproduction Society v EMI* (1958) 32 A.L.J.R.ALJR 306.

66 Nairn (n 11) ‘The Twilight of the British State’ fn 27.

67 Paolo Carozza argues that ‘Sovereignty … is conceptually contradictory to subsidiarity as a way of organizing relations among different levels of authority. Where sovereignty draws binary lines … subsidiarity entails a set of fluid, interconnected, and conditioned layers between the individual person and the most comprehensive community, in which the boundaries of state sovereignty are always only provisional and permeable’: Carozza (n 27) 62.

68 Roxana Banu, ‘Constructing Imperial Authority: The Intersection of British Imperial Constitutional Law and Private International Law’ OJLS (forthcoming).

69 Martin Loughlin, ‘The British Constitution and the Irish Question’ (2024) 35 (3) KLJ 395, 395.

70 R Brazier, ‘The Constitution of the United Kingdom’ (1999) 58(1) CLJ 96, 100.

71 Feldman (n 34) 346.

72 There is an extensive literature on the relationship between the UK government and the devolved governments since the UK left the EU. See eg Oran Doyle, Aileen McHarg and Jo Murkens, *The Brexit Challenge for Ireland and the United Kingdom* (CUP 2021); McCrudden, *The Law and Practice of the Ireland–Northern Ireland Protocol* (CUP 2022); C McCorkindale, ‘Legislative Consent, Constitutional Convention, and Constitutional Ill-Health’ (2024) 75(1) NILQ 83. A key argument in that literature is that the Brexit process, accompanied by the Internal Market Act and the Northern Ireland Protocol, has restored unitary central control to Whitehall. I will not recapitulate that argument here. Rather, I wish to explain how the retrenchment of central control exploits structures which long pre-date Brexit.

73
*Treatment of Polish Nationals and Other Persons of Polish Origin or Speech in the Danzig Territory* (Advisory Opinion) 1932, PCIJ Series A/B, No 44, 24. The classic exposition of this principle is the Alabama Arbitration (1872) 4 Moore International Arbitrations 4144, 4156–7. I am grateful to Eirik Bjorge for this example.

74 The king provides a visible example of this connection, and not merely in an airily symbolic way. When Charles visits a state such as Australia, of which he is king, he takes advice from the Australian government, not from the UK government through the Foreign Secretary. Nor is he required to take advice when addressing the Commonwealth, of which he is the head. Charles’s speech at the Commonwealth Heads of Government Meeting in Samoa in 2024, and especially its references to ‘painful aspects’ of Britain’s past, opened a debate about legal responsibility for slavery, a crime against humanity with important implications for the foreign policy of those states and potentially for their legal obligations to each other. The UK government sought to shut this debate down.

75
*Halsbury’s Laws of England* (Sweet & Maxwell 2021); *Attorney-General of New Zealand v Ortiz and Others* [1982] 3 WLR 570, 20.

76
*Sue v Hill* (1999) 199 CLR 462; *A-G (United Kingdom) v Heinemann Publishers Australia Pty Ltd* (1988) 165 CLR 30.

77 Kilbrandon Commission (n 18) 1381.

78 Interpretation Act 1978, s 5, sch 1.

79 See eg Investigatory Powers Act 2016, s 13(2).

80
*Halsbury’s Laws* (n 75) 602.

81 See *Jersey Fishermen’s Association v States of Guernsey* [2007] UKPC 30, 239. cf *Royal Society v Robinson* [2015] EWHC 3442 (Ch).

82
*Jersey Fishermen’s Association* (n 81).

83
*Stoneham v The Ocean, Railway, and General Accident Insurance Company* (1887) 19 QBD 237.

84
*Partington v Rossiter* [2021] EWCA Civ 1564.

85 [1962] 1 WLR 173.

86 ibid 177, 178. The accident in Ringrose took place in the channel, between England and the Channel Islands.

87
*Partington* (n 84) [24].

88 AV Dicey, *The Law of Domicile as a Branch of the Law of England* (Stevens, 1879) 33.

89 T Frost and CRG Murray, ‘Homeland: Reconceptualising the Chagossians’ Litigation’ (2020) 40 (4) OJLS 764 fn 16.

90 See Banu (n 68).

91 Donal Coffey ‘1916, 1921 and the Destruction of the Legal Unity of the British Empire’ (2016) 39(2) DULJ (NS) 334.

92
*Campbell v Hall* (1774) 1 Cowper 204, 98 ER 1045; *Sammut v Strickland* [1938] AC 678.

93 Kilbrandon Commission (n 18) 411, as quoted in Nairn (n 12) ‘The Twilight of the British State’ fn 38. The Commissioners recorded there that they ‘do not doubt that more logical and orderly races would have swept all these away long ago’. Nevertheless, they ‘have not approached the Islands in any spirit of reforming zeal’.

94 Ian Hendry and Susan Dickson, *British Overseas Territories Law* (Bloombsury 2011) 1.

95 Anthony King, *The British Constitution* (OUP 2009). I am grateful to Colin Murray for this example.

96
*MacCormick v Lord Advocate* 1953 SC 396, 411 (Lord President Cooper); *Gibson v Lord Advocate* 1975 SC 136 (Lord Keith).

97
*Lord Gray’s Motion* 2000 SC (HL) 46, [2002] 1 AC 124, 138.

98 RK Murray, ‘The Anglo Scottish Union’ *Scots Law Times* (4 November 1961); MacCormick (n 15) ch 4.

99 MacCormick (n 15) 58.

100 See especially *Reference by the Lord Advocate* [2022] UKSC 31; *Re: Allister* [2023] UKSC 5.

101 Martin Loughlin, ‘The British Constitution and the Irish Question’ (2024) 35 (3) KLJ 395, 395.

102 ibid 400.

103 ibid 415.

104 ibid 399.

105 ibid 411.

106 See eg the Constitutional Reform and Governance Act 2010, s 5. The *Welsh* civil service has been organised on an essentially separate basis since The Troubles.

107 Loughlin (n 101) 403.

108 Loughlin claims ‘no mainstream British political parties organise and stand for election in Northern Ireland’, ibid 411. This is not quite correct. The Conservative Party has stood in Northern Ireland since the 1980s, albeit not very successfully.

109 ibid 400.

110 We should underline, with Loughlin, the effective disenfranchisement of the Catholic community in Northern Ireland long after universal suffrage in Great Britain.

111 England did.

112 Linda Colley, *The Gun, the Ship and the Pen: Warfare, Constitutions and the Making of the Modern World* (Profile Books 2021).

113 See Hendry and Dickson (n 94) 14.

114 Dicey, *Introduction* (n 36) 136. Dicey also said ‘The constitution indeed of a Dominion in general originates in and depends upon an Act, or Acts, of the Imperial Parliament; and these constitutional statutes are assuredly liable to be changed by the Imperial Parliament’.

115 See eg Scotland Act, s 28(7), which says ‘this section does not affect the power of the Parliament of the United Kingdom to make laws for Scotland’ is a reference to the legal power of the UK Parliament.

116 Kilbrandon Commission (n 18) 1473.

117 See the caveat at n 58.

118 See SAMLA, s 51(2).

119 See eg Scotland Act, s 28(7), which says ‘this section does not affect the power of the Parliament of the United Kingdom to make laws for Scotland’ is a reference to the legal power of the UK Parliament.

120 House of Commons Justice Committee, *Crown Dependencies: Developments since 2010* (2013, HC 726) 20; David Torrance, ‘The Crown Dependancies’ (2025) Commons Library Research Briefing No CBP8611, 3.1.

121 Torrance (n 120) 3.2.

122
*Madzimbamuto v Lardner-Burke and George* [1969] 1 AC 645.

123 Ibid 722

124
*R (Barclay) v Secretary of State for Justice* [2014] UKSC 54 [12]

125 There was an English constituency in Calais in the 16th century. The Channel Islands were invited to send representatives to Westminster in the 17th and 18th centuries, though the invitation was ignored. On the recent reconfiguration of the Manx legislature, see PW Edge, ‘Lisvane’s Legacy? Constitutional Reform in the Isle of Man’ (2020) 40 (1) LS 22.

126
*Halsbury’s Laws* (n 75) [617].

127 John Finnis, ‘Common Law Constraints: Whose Common Good Counts?’ (2008) Oxford Legal Studies Research Paper No 10/2008.

128 MacCormick (n 15) 28. Maitland, Constitutional History of England (Cambridge, CUP 1908) 418

129 The last time this power was exercised in respect of Dependencies was the Court of Appeal (Channel Islands) Order 1949. See *Barclay* (n 124).

130 Torrance (n 120) 1.4.

131 House of Commons Justice Committee (n 120) 16.

132 Torrance (n 120) 1.4.

133 Newfoundland is an example.

134 Philip Loft, ‘The Potential for Direct Rule in the British Virgin Islands’ (2022) Commons Library Research Briefing No 9538. For background see *Misick v. R* [2015] UKPC 31

135 See generally Paul Scott, ‘Corruption and the Constitutional Position of the Overseas Territories’ (2023) 23(1) OUCLJ 44.

136 Barber (n 1). cf Larsen (n 21) 19.

137 Burma left the Commonwealth in 1948. Some members, such as Mozambique, were never British colonies.

138 Inter-Imperial Relations Committee, *Report of Inter-Imperial Relations Committee of the Imperial Conference* (Cmd 2768, 1926).

139 V. Bogdanor, *The Sovereign and the Commonwealth in The Monarchy and the Constitution* (Oxford, Oxford University Press 1997) at 247.

140
*Quark Fishing* (n 14).

141 Donal Coffey, ‘British, Commonwealth, and Irish Responses to the Abdication of King Edward VIII’ (2009) 44 Irish Jurist 95.

142
*R (Indian Association of Alberta) v Secretary of State for Foreign and Commonwealth Affairs* [1982] QB 892, 917 (Lord Denning).

143 ibid 922. The passage is a quote from *Halsbury’s Laws* (n 75).

144 On which see Peter Lindseth, ‘The Metabolic Constitution and the Limits of EU Legal Pluralism’ in Gareth Davies and Matej Avbelj (eds), *Research Handbook on Legal Pluralism and EU Law* (Edward Elgar Publishing 2018).

145 See n 14.

146 ibid [10].

147
*R (Bancoult) v Secretary of State for Foreign and Commonwealth Affairs (No 2)* [2008] UKHL 61.

148 ibid [49].

149 ibid [94].

150 Anne Twomey ‘Responsible Government and the Divisibility of the Crown’ 2008 PL 742, 764.

151 There is a historical argument that Scotland has a separate Crown and that there is a separate Crown ‘in right of the Scottish Administration’. See Scotland Act 1998, s 99. I need not pursue that argument here for reasons Twomey explains. The reference is to the Crown in the sense of an executive government, not in the sense of the public interest.

152 Twomey (n 150) 766.

153 Scotland Act, s 46.

154 Meanwhile, each devolved government has Interests Sections abroad. These offices represent the interests of the devolved government, often sitting within a British Embassy or High Commission, but with separate reporting lines.

155 ibid 557.

156 Barber (n 1). cf Larsen (n 21) 19.

157 Dicey, *England’s Case Against Home Rule* (n 36) 248.

158 Hansard, Vol 299 (31 July 1997) Column 468.

159 ibid 169–70.

160 Bryce (n 29).

